# A two-day-old hyperthyroid neonate with thyroid hormone resistance born to a mother with well-controlled Graves’ disease: a case report

**DOI:** 10.1186/1752-1947-6-246

**Published:** 2012-08-20

**Authors:** Shuichi Yatsuga, Yuji Hiromatsu, Shigekazu Sasaki, Hirotoshi Nakamura, Koju Katayama, Junko Nishioka, Yasutoshi Koga

**Affiliations:** 1 Department of Pediatrics and Child Health, Kurume University School of Medicine, 67 Asahi-Machi, Kurume, Fukuoka, 830-0011, Japan; 2 Department of Medicine, Division of Endocrinology and Metabolism, Kurume University School of Medicine, 67 Asahi-Machi, Kurume, Fukuoka, 830-0011, Japan; 3 Department of Internal Medicine, Hamamatsu University School of Medicine, 1-20-1 Handayama, Hamamatsu, Shizuoka, 431-3192, Japan

**Keywords:** Hyperthyroid symptoms, Maternal Graves’ disease, Symptomatic neonate with resistance to thyroid hormone, Treatment for resistance to thyroid hormone

## Abstract

**Introduction:**

Resistance to thyroid hormone is a syndrome caused by thyroid hormone receptor β mutations, which are usually inherited in an autosomal-dominant pattern.

**Case presentation:**

Our patient, a Japanese neonate boy, showed hyperthyroid symptoms at age two days. Although our patient was diagnosed as having resistance to thyroid hormone, his hyperthyroid symptoms continued for two weeks. Therefore, our patient was treated with methimazole and iodine for two weeks from birth, showing no side effects and no symptoms upon treatment. At age 70 days, an R243W mutation in thyroid hormone receptor β was detected in our patient; while absent in his mother, the mutation was present in his father, who never showed any symptoms.

**Conclusions:**

To the best of our knowledge this is the first case report of a resistance to thyroid hormone in a neonate presenting with hyperthyroid symptoms born to a mother with Graves’ disease and treated with methimazole and iodine. These results suggest that methimazole and iodine may be a good short-term option for treatment.

## Introduction

Resistance to thyroid hormone (RTH) is an autosomal dominant (AD) syndrome in which an individual’s response to thyroid hormone (TH) is decreased due to mutations in the TH receptor β gene (*TRβ*) [1,2]. Patients with RTH have increased serum TH levels and increased or normal thyroid-stimulating hormone (TSH) levels. The clinical characteristics of RTH vary strikingly, as even the characteristics of various tissues within the same individual or family members who carry identical mutations differ notably [3]. While most patients are asymptomatic, some are symptomatic and show main clinical features such as goiter, hyperactivity, and tachycardia [1]. However, although RTH has been well investigated recently, the response of patients with RTH to treatment remains unclear.

A neonate born to a mother with Graves’ disease (GD) has an increased risk of developing neonatal GD, a rare condition that affects 1% to 5% of babies born to mothers who have hyperthyroidism during pregnancy. Most babies are asymptomatic because the mother normally receives treatment for her GD. In contrast, patients with RTH do not usually receive treatment because they show no symptoms. Indeed, even when they do show symptoms, the results of treatment have been discouraging. Kim *et al.* treated one symptomatic patient with RTH with methimazole (MMI) and T_4_ treatment but had to cease treatment because a large goiter developed [4], improving upon treatment withdrawal.

Here, we describe a symptomatic neonate with an R243W *TRβ* mutation inherited from his non-symptomatic father. Our patient, who was born to a mother with well-controlled GD, continued to show hyperthyroid symptoms for two weeks, at which point we administered MMI and iodine for another two weeks and monitored our patient’s symptoms and thyroid function tests.

## Case presentation

Our patient’s parents were non-consanguineous and of Japanese origin, with an unremarkable family history except for the mother, who had thyroid symptoms. The mother had an onset of GD at 23 years of age and was subsequently treated for hyperthyroidism with 30mg/day MMI and 50mg/day iodine potassium. After two weeks of treatment, our patient’s mother experienced side effects from the MMI, and the regimen was therefore changed to 300mg/day of propylthiouracil (PTU). The PTU dose was then reduced gradually as thyroid hormone levels improved. The mother became pregnant seven months after the GD diagnosis and was treated with 50mg PTU every two days. Thyroid hormones and antibodies related to GD in the mother were within normal ranges throughout the pregnancy (Table 1).

**Table 1:** Thyroid hormone profile of our patient and his mother and father

**Profile and normal range**	**Onset**	**P0**	**P5**	**P8**	**Delivery**	**Patient’s father**	**Patient**
TSH (μIU/mL) (0.4 to 4.0)	0	0	0.61	0.27	0.04	0.59	5.38 (1.0 to 38.9)
FT_4_ (ng/dL) (0.8 to 1.9)	5.64	1.53	0.83	0.9	1.16	2.87	4.76 (2.0 to 4.9)
FT_3_ (pg/mL) (2.2 to 4.1)	22.38	2.65	1.93	2.27	NA	4.88	6.7 (2.0 to 6.1)
TgAb (IU/mL) (<28)	0.3	0.3	0.3	0.3	NA	0.3	<0.1 (<28)
TPOAb (IU/mL) (<16)	9.1	0.7	0.3	0.3	NA	<0.3	<0.1 (<16)
TRAb (IU/L) (<1)	15	4	1.2	1	NA	<0.1	<0.1 (<1)
TSAb (%) (<180)	188	239	135	132	NA	130	173 (<180)
Tg (ng/mL) (<32.7)	540	130	68	NA	NA	NA	NA

Thyroid hormone profile of the mother (onset of GD to child delivery), the father, and our patient at two days after birth. P0 is zero months’ pregnant, P5, five months’ pregnant, P8, eight months’ pregnant. Values at onset and P0 indicate typical Graves’ disease (GD). GD was well controlled during pregnancy by propylthiouracil. Reference values for adults are used for the mother and the father; reference values for neonates are used for our patient. In the father and our patient, all values for thyroid antibodies were negative, indicating resistance to thyroid hormone. Thyroid-stimulating antibody (TSAb) was measured using radioimmunoassay (RIA), while all other values were measured using electrochemiluminescence immunoassay (ECLIA).

FT_3_/T_4_, free T_3_/T_4_; Tg, thyroglobulin; TgAb, thyroglobulin antibody; TPOAb, thyroid peroxidase antibody; TRAb, TSH receptor antibody; NA, not available.

Our patient was born at 38 weeks into the pregnancy following a non-problematic gestation period. His birth weight was 2910g. Our patient exhibited visible hyperthyroid symptoms two days after birth, including tachycardia, frequent bowel movements, and hyper-irritability. A complete blood cell count and blood chemistry examination revealed normal levels with the exception of increased thyroid hormone levels (Table 1). Antibodies associated with thyroid disease were within normal ranges (Table 1). Electrocardiography primarily showed a regular sinus rhythm, and our patient’s sleeping heart rate was slightly elevated at 150 to 160 beats per minute compared with the normal range of 120 to 140 beats per minute. Ultrasonography revealed the thyroid to be normal in size with no nodules. A TSH-secreting adenoma (TSHoma) was ruled out through magnetic resonance imaging (MRI) scans of the pituitary gland, and our patient’s human chorionic gonadotropin β (hCG-β) levels were found to be normal at admission. Our patient was suspected of having RTH rather than neonatal GD due to unsuppressed TSH and high free T_4_ (FT_4_) and T_3_ (FT_3_) levels.

**Figure 1:**
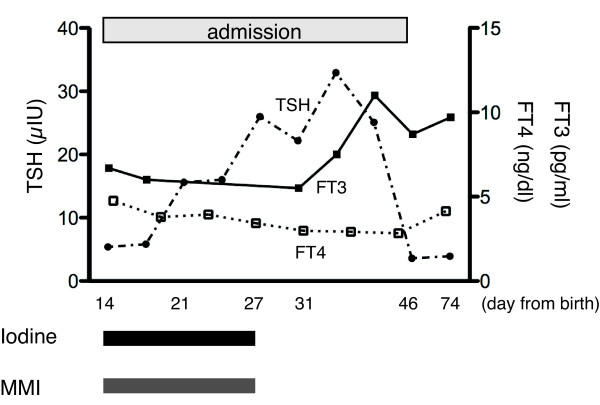
**Thyroid hormone and treatment course of our patient during and after admission.** After increasing methimazole (MMI) and iodine dosage, thyroid-stimulating hormone (TSH) levels were increased while those of free T_4_ (FT_4_) and FT_3_ were decreased. When MMI and iodine were not administered, TSH fell back to normal levels, while FT_4_ and FT_3_ increased. Administered iodine and MMI doses are shown in black and gray squares, respectively.

At 14 days old, our patient still continued to show hyperthyroid symptoms and was therefore treated with 0.65mg/kg/day MMI and 12.6mg/day iodine. Our patient responded to the therapy clinically. His irritability diminished, and his sleeping heart rate reduced to 130 to 150 beats per minute. Expectedly, his TSH level increased, and his FT_4_ and FT_3_ levels decreased (Figure 1). During this course, our patient presented no elevation of antibodies related to GD, and an abnormal thyroid hormone profile continued without goiter. MMI and iodine were discontinued at age 28 days, as our patient’s symptoms, particularly hyper-irritability and frequent bowel movements, were improved. In the two-week treatment period, our patient showed neither severe nor worsening symptoms. Our patient was hospitalized for a total of 33 days before being discharged, at which point our patient was confirmed to be euthyroid; follow-up was conducted every three months using thyroid function tests. Today, our patient is three years old and remains clinically euthyroid without the use of therapeutic drugs after discharge. Our patient has also reached developmental milestones appropriate for his age.

Our patient’s bone development was also normal, and his electrocardiography, Holter electrocardiography, and echocardiogram findings were all within normal limits, with no severe cardiac complications observed. Our patient’s father had never experienced symptoms of hypothyroid or hyperthyroidism, although inappropriate thyroid hormone levels were seen in laboratory tests (Table 1).

Blood samples were obtained from our patient and both his parents, and genomic deoxyribonucleic acid (DNA) was isolated from leukocytes using standard protocols. Sequencing the patient’s *TRβ* gene revealed a missense mutation that causes an R243W substitution within the receptor’s T_3_-binding domain. While the mother’s *TRβ* gene did not contain this mutation, the father’s did.

## Discussion

This case report describes a baby boy with a *TRβ* R243W mutation born to a mother with no RTH mutation and with no family history of RTH. Our patient’s asymptomatic father carried the same mutation as our patient.

The R243W point mutation, first detected by Pohlenz *et al.*[5], has a mechanism of action differing from that of other *TRβ* mutations, as the R243W receptor has normal T_3_-binding affinity but transactivates poorly upon binding T_3_, thereby conferring a dominant-negative effect [6,7]. The resulting phenotype is usually euthyroid with occasional hypothyroidism being observed. Regardless, clinical features of both hypothyroidism and hyperthyroidism are expected due to variable resistance in the different tissues of an individual. In the present case, a small amount of PTU from the mother transmitted to the fetus may have induced hyperthyroidism just after birth. Since such quantities of PTU may slightly suppress thyroid hormones in the fetus, a sudden release of thyroid hormones after birth may have occurred, causing our patient to be initially diagnosed as having hyperthyroidism.

The clinical phenotype for R243W also differs among families and individuals. In fact, the same mutation can cause either generalized RTH or pituitary RTH in different individuals within the same family. For example, a boy who had slight attention-deficit hyperactivity disorder and the R243W mutation was born to a mother who remained clinically euthyroid with the same mutation [8]. Additionally, only a weak correlation has been observed between a given mutation and the development of RTH [1,3,9]. In rare cases, RTH coexists with GD [10,11]. Considering the mother’s condition in the present case, it would be normal for our patient to be suspected of having neonatal GD. However, since our patient showed no suppressed TSH levels and had high FT_4_ and FT_3_ levels with no antibodies related to GD, our patient was diagnosed as having RTH.

Patients with RTH are not usually treated because many patients do not have significant symptoms. Kim et al. administered MMI plus T_4_ treatment for a symptomatic 11-month-old patient who had hyperthyroidism secondary to RTH [4]. However, a large goiter developed without clinical improvements, and after withdrawing treatment, the goiter then improved. In the present case, we treated our patient with MMI and iodine for two weeks, after which our patient showed improved hyperthyroid symptoms and no goiter. In the report by Kim *et al.*, the patient had severe symptoms, including a failure to thrive, verbal delays and tachycardia. Our patient had symptoms of tachycardia, diarrhea, and hyper-irritability but showed no failure to thrive or developmental delay, an aspect we attribute to our treatment program.

The differential diagnoses in the present case were neonatal GD and TSHoma. TSHoma can be discovered relatively easily using magnetic resonance imaging (MRI); however, when a pregnant mother has GD, a neonate with hyperthyroid symptoms is typically expected to have neonatal GD, which can often lead to a misdiagnosis of RTH [10]. Consulting previous papers, which showed how to diagnose RTH with thyroid diseases [12,13], would have avoided such a misdiagnosis.

## Conclusions

We describe the case of a neonate presenting with hyperthyroid symptoms. In hyperthyroid neonates born to mothers with GD, it is important to examine the thyroid hormone levels of both parents. Had serum TSH receptor antibody (TRAb) or thyroid-stimulating antibody (TSAb) been elevated in both our patient and his mother, diagnosis of RTH would have been further delayed. To the best of our knowledge this is the first report of a symptomatic neonate with RTH born to a mother with GD and treated by MMI and iodine in the neonatal period without side effects. MMI and iodine, therefore, may make for optimal short-term treatment in hyperthyroid RTH neonates.

## Consent

Written informed consent was obtained from the patient’s next-of-kin for publication of this case report and any accompanying images. A copy of the written consent is available for review from the Editor-in-Chief of this journal.

## Competing interests

The authors report no financial competing interests.

## Authors’ contributions

SY, YH, KK, JN, and YK examined our patient and discussed the diagnosis and treatment. SS and HN carried out the molecular genetic study, and SY drafted the manuscript. All authors read and approved the final manuscript.
